# Empagliflozin induces apoptotic-signaling pathway in embryonic vasculature: *In vivo* and *in silico* approaches *via* chick’s yolk sac membrane model

**DOI:** 10.3389/fphar.2022.970402

**Published:** 2022-09-01

**Authors:** Saeedeh Mosallanejad, Mehdi Mahmoodi, Hadi Tavakkoli, Ahmad Khosravi, Ehsan Salarkia, Alireza Keyhani, Shahriar Dabiri, Mohammad Hossein Gozashti, Abbas Pardakhty, Hadi Khodabandehloo, Hossein Pourghadamyari

**Affiliations:** ^1^ Department of Clinical Biochemistry, Afzalipour School of Medicine, Kerman University of Medical Sciences, Kerman, Iran; ^2^ Department of Clinical Sciences, School of Veterinary Medicine, Shahid Bahonar University of Kerman, Kerman, Iran; ^3^ Leishmaniasis Research Center, Kerman University of Medical Sciences, Kerman, Iran; ^4^ Afzalipour School of Medicine, Pathology and Stem Cell Research Center, Kerman University of Medical Science, Kerman, Iran; ^5^ Endocrinology and Metabolism Research Center, Institute of Basic and Clinical Physiology Sciences, Kerman University of Medical Sciences, Kerman, Iran; ^6^ Pharmaceutics Research Center, Kerman University of Medical Sciences, Kerman, Iran; ^7^ Department of Clinical Biochemistry, School of Medicine Zanjan University of Medical Sciences, Zanjan, Iran; ^8^ Gastroenterology and Hepatology Research Center, Institute of Basic and Clinical Physiology Sciences, Kerman University of Medical Sciences, Kerman, Iran

**Keywords:** empagliflozin, apoptosis, yolk sac membrane, immunohistochemistry, molecular dynamics

## Abstract

The present investigation was conducted to evaluate the vascular-toxicity of empagliflozin (EMP) in embryonic vasculature. Firstly, the vascular-toxicity of the drug as well as its interaction with apoptotic regulator proteins was predicted *via in silico* approach. In the next step, the apoptotic-signaling pathway in embryonic vasculature was evaluated using a chick’s YSM model. *In silico* simulation confirmed vascular-toxicity of EMP. There was also an accurate affinity between EMP, Bax and Bcl-2 (−7.9 kcal/mol). Molecular dynamics assay revealed complex stability in the human body conditions. Furthermore, EMP is suggested to alter Bcl-2 more than BAX. Morphometric quantification of the vessels showed that the apoptotic activity of EMP in embryonic vasculature was related to a marked reduction in vessel area, vessel diameter and mean capillary area. Based on the qPCR and immunohistochemistry assays, enhanced expression level of BAX and reduced expression level of Bcl-2 confirmed apoptotic responses in the vessels of the YSM. We observed that induction of an apoptotic signal can cause the embryonic defect of the vascular system following EMP treatment. The acquired data also raised suspicions that alteration in apoptotic genes and proteins in the vasculature are two critical pathways in vascular-toxicity of EMP.

## 1 Introduction

Empagliflozin (EMP) is in a class of medications named sodium-glucose co-transporter 2 (SGLT2) inhibitor. Inhibition of the SGLT2 lowers blood glucose through an insulin-independent mechanism (Salukhov et al., 2020). The EMP is a novel drug for improving glycemia in adults with type 2 diabetes mellitus *via* increasing urinary glucose excretion (Frampton, 2018). The drug is prescribed in diabetic patients as monotherapy or in combination with other glucose-lowering medicinal products. It is also applied to decrease the risk of stroke, heart attack and death in people who have type 2 diabetes along with heart disease. Furthermore, its advantages such as the low risk of hypoglycemia and absence of weight gain support its consideration as a suitable medication for patients with type 2 diabetes mellitus (Frampton, 2018; Ferreira et al., 2022; Li et al., 2022; Tuttle et al., 2022).

Various studies in the literature focus on the toxicity and side effects following EMP treatment (Bogdanffy et al., 2014; Halimi and Vergès, 2014; Taub et al., 2015; Neeland et al., 2016; Knight et al., 2018; Cianciolo et al., 2020). The toxic effect of drugs is of great concern in pregnant women. It is well proven that following consumption, EMP readily passes the placenta and enters the fetal circulation (Neeland et al., 2016; Formoso et al., 2018). In addition, the drug is categorized in group D of the Australia TGA pregnancy category and it is suspected to have adverse pharmacological effects. In the United States Food and Drug Administration (FDA) pregnancy category, there is insufficient data on pregnant women to determine the adverse effects of EMP on the fetus. However, additional research is required to assess the adverse effects of EMP and its damage to fetal growth.

Although the increasing prescription and consumption of antidiabetic compounds is expected in some regions of the world, little data has been published in the literature about the toxicity and side effects of EMP for fetuses and embryonic vessels. Furthermore, the exact pathways by which EMP affects embryonic vessels are not clear. One of the most significant pathways influencing the fetus and embryonic vessel is apoptosis (Haanen and Vermes, 1996; Singh et al., 2019; Salari et al., 2021). Consequently, we hypothesized that EMP induces apoptotic-signaling pathways in embryonic vasculature and causes vascular-toxicity. So far, this probable vascular apoptotic activity of EMP has not been assessed. The current experiment was conducted to address the subsequent questions:

a) Does EMP cause alteration in the fetus vasculature?
b) Does EMP alter the gene expression and proteins, which have critical functions in vascular apoptosis?
c) How is the binding affinity among EMP and apoptotic proteins?

To clarify those questions, a chick’s yolk sac membrane (YSM) model was employed and *in silico* study was done to assess the apoptotic effect of EMP. In this respect, the interactions between EPM and apoptotic proteins (BAX and Bcl-2) were evaluated by *in silico* investigation. The acquired data have been also accompanied by *in vivo* experiment, molecular and immunohistochemistry techniques to assess the impact of EPM on the YSM vasculature.

## 2 Material and methods

### 2.1 (A) Vascular-toxicity of empagliflozin using in silico prediction

**FIGURE 1:**
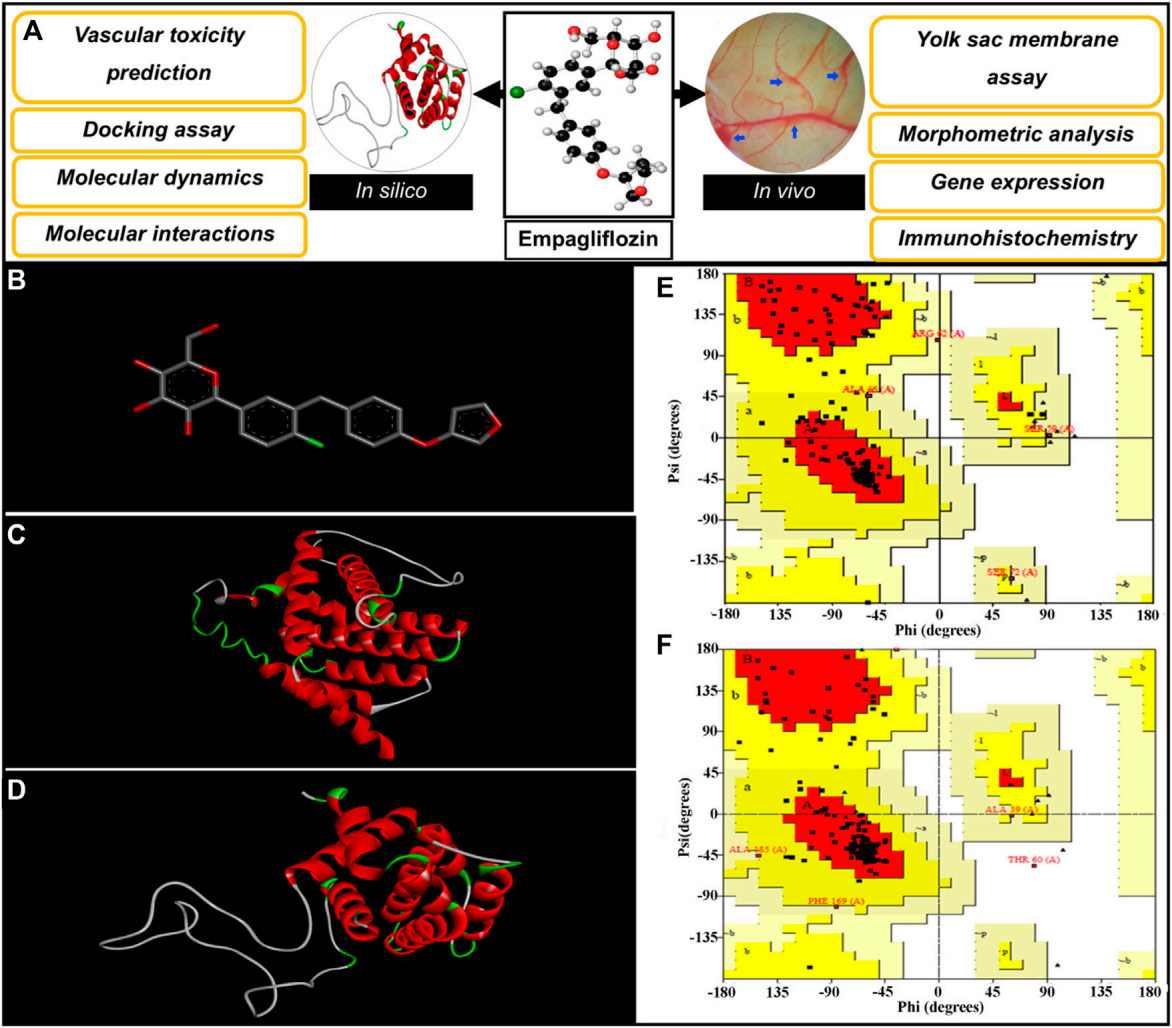
**(A)** Diagrammatic representation of the steps involved in investigating the vascular-toxicity of EMP in embryonic vasculature. **(B)** Structure of EMP. **(C,D)** 3-D Conformations of constructed BAX and Bcl-2, respectively; *Gallus gallus*. **(E,F)** Ramachandran plots for simulated BAX and Bcl-2.

Various programs and databases were used to predict the vascular-toxicity of EMP. The steps are presented in Figure 1A. The interaction of EMP with proteins, which are associated with apoptosis in embryonic vasculature, was also accessed *via* docking and molecular dynamics simulation. In this regard, the EMP structure was acquired from the PubChem server (https://pubchem.ncbi.nlm.nih.gov/, CID: 11949646) (Figure 1B). The obtained structure was sent to the PASS server to investigate the toxic effect of the molecule (http://www.pharmaexpert.ru/passonline/). The PASS server has been used to predict the toxicity of various agents (Poroikov et al., 2007; Lagunin et al., 2011; Agahi et al., 2020; Salari et al., 2021). The toxic effect and Pa (probable to be active) were monitored.

#### 2.1.1 Docking assay

The possible interactions of EMP with apoptotic proteins (Bax and Bcl-2) were evaluated *via* docking assay. The structural sequences of Bax and Bcl-2 proteins (*Gallus gall*us, ID: ACR83547.1 and NP_990670.2) were gained from the GenBank, NCBI server and proper templates were selected by the blasting tool of the NCBI server (https://blast.ncbi.nlm.nih.gov/Blast.cgi). The Protein Data Bank (PDB) server (https://www.rcsb.org/) was selected as a database for blasting. In the following step, the adjacent homolog conformations of proteins were selected to serve as conformational templates. The SWISS-MODEL (https://swissmodel.expasy.org/) server was used for structural simulations of BAX and Bcl-2 proteins based on the user template structures (Waterhouse et al., 2018). The generated models were further assessed by Ramachandran plot from PROCHECK (http://servicesn.mbi.ucla.edu/Verify3D/) (Gopalakrishnan et al., 2007).

Ligand and receptors were adjusted for docking assay by adjusted charges (partial) and atoms (hydrogen). The docking was performed by AutoDock Vina to clear the binding affinity of EMP to Bax and Bcl-2 proteins (Trott and Olson, 2010). A specific grid box was performed with 54 × 40 × 40 values and the resolution of 1Å, to cover the whole structure. Finally, the binding energies were monitored and the best conformations (lowest energy) were chosen.

#### 2.1.2 Molecular dynamics simulation

Molecular dynamics (MD) simulations were done by Gromacs 5.4.1 and the amber force field (Wang et al., 2004; Van Der Spoel et al., 2005). The GROMACS package was applied for molecular dynamics simulations by various researchers (Jacob et al., 2017; Tavakkoli et al., 2021; Zhang et al., 2022). The docked structures of the EMP with Bax and Bcl-2 were placed inside a dodecahedron box and were solvated withTIP3P water molecules. The atomic charges of structures were analyzed, and appropriate ions (Na or Cl) were used to maintain the system’s neutrality based on electrical charges. Electrostatic interactions were simulated using particle mesh Ewald and cut-off (short-range) distance algorithms (Essmann et al., 1995). The van der Waals energy was considered as 1 nm. All molecular bonds were constrained *via* the LINCS algorithm at equilibrium distance (Hess et al., 1997). The energy of the system was minimized by steepest descent assay (Lindahl et al., 2001). The pressure and temperature of the MD simulations were set at 1 bar and 310.65 K (37.5°C) *via* the Parrinello-Rahman algorithm. The MD simulation was also done to identify the stabilities between EMP, BAX and Bcl-2 proteins inside the human body conditions (aqueous environment, 37.5°C). The trajectory data were analyzed *via* Root-mean-square fluctuation (RMSF), solvent accessible surface area (SASA), do_dssp, and binding free energy (Kumari et al., 2014; Zhang and Sagui, 2015; Valdés-Tresanco et al., 2021). For computing the affinity of EMP with Bax and Bcl-2, the binding free energies were measured *via* the MM-PBSA assay (Kumari et al., 2014; Rifai et al., 2019). The energies including van der Waals, electrostatic, polar solvation and non-polar solvation were considered for calculating free energy.

### 2.2 Apoptotic-signaling pathway in embryonic vasculature following empagliflozin treatment

#### 2.2.1 Drug and eggs

The EMP was obtained from Abidi Pharmaceutical Co., Iran. The dosage of (10 mg/kg of egg weight which is the same as the applied dosage in humans) was selected for evaluating the effect of the drug on embryonic vasculature (Frampton, 2018; Ferreira et al., 2022). Fertilized chicken eggs (Cobb 504) were taken from Mahani Broiler Co., Iran. The fertilized eggs were immediately incubated at an appropriate temperature and relative humidity (37.5°C and 60% humidity).

#### 2.2.2 Yolk sac membrane assay

The eggs were separated into two treatment groups containing 10 fertilized eggs in each. In the first group (control group), the fertilized eggs were treated with sterilized phosphate-buffered saline (PBS). The fertilized eggs of group 2 were treated with EMP in the same way. The EMP or sterilized PBS was at three different time points (24, 48, and 72 h following the growing period; to embryonic development stages of 6, 12, and 20 according to the Hamburger and Hamilton developmental stages, respectively) (Hamburger and Hamilton, 1951). A pinpoint widow of 1 mm was drilled in the eggs (outer shell and shell membrane) into the air cell of the egg and the embryos were treated with direct inoculation on the internal shell membrane *via* a 50 ml syringe, Hamilton tool. This procedure of inoculation has been applied before (Oosterbaan et al., 2012; Khosravi et al., 2019). After injection, the widow was closed with melted paraffin (Merck CO., Germany) and the embryonated eggs were returned to the incubator for better development. On the fourth day of the incubation period, a visualized window of 20 mm × 20 mm was opened in the eggs to capture the YSM vasculature. High-quality captures with the resolution of 3,000 × 4,000 pixels and 10X magnification were acquired *via* a stereozoom microscope (Luxeo stereo-microscope; Labomed; United States) supported by a Canon camera, 750-EOD.

#### 2.2.3 Morphometric analysis of the yolk sac membrane vasculature

The Fiji 1.53 (the United States National Institutes of Health; United States), MATLAB (Mathworks, Natick, MA, United States) and Digimizer 5.3.4 programs (Med Calc Software bvba, Belgium), were applied to analyze the vascular system (Kovesi, 2004; Schindelin et al., 2012; Khosravi et al., 2022). In Brief, the YSM images were loaded and the unique areas (1900 × 2,850 pixels) were selected in TIFF file format. The unique vascular area was considered exactly to the right corner of the anterior and posterior vitelline vein. In the next step, image thresholds were enhanced *via* the CLAHE algorithm to optimize vessel detection. To extract the vascular areas from non-vascular, the image type was converted to an 8-bit binary format to apply a specific color quality to all pixels. At last, binary images were skeletonized.

#### 2.2.4 Measurements

The percentages of the vessel area and average vessel diameter (mm) were measured from the improved images. Furthermore, the mean capillary area (MCA) was measured. To accomplish this goal, the fields containing no branch vessels were chosen in images. Five mentioned fields per image were selected and the mean percentage of the fields including black pixels was computed. The black pixels point toward the red color (e.g. blood) in the original pictures. The average of all fields calculated in each picture is described as MCA (Seidlitz et al., 2004).

#### 2.2.5 Gene expression

The RNA from the YSM vasculature was extracted and cDNA synthesis was performed on 500 ng RNA *via* RT reagent kit (TaKaRa Clontech, Japan) based on the manufacturer’s instructions. The sample quality was evaluated (ND-1000, Thermo Scientific Wilmington, DE, United States) and qPCR reaction was made by the SYBR Green procedure (SYBR Premix Ex Ta II; Japan). The sequences of reference genes and primers are described previously (Khosravi et al., 2022) and presented in S4. Finally, the impact of EMP on the apoptotic-regulator genes (Bax and Bcl-2) was evaluated.

#### 2.2.6 Immunohistochemistry assay

The YSM vasculatures were carefully fixed into buffered formalin 10%. Then, the fixed samples were embedded in blocked paraffin for tissue preparation. The IHC assay was done for Bax and Bcl-2 markers using (Zytomed, ID code: 502_17990, Germany) and (monoclonal antibody, mouse, ID code: PDMO16- lot_H147, American) based on the manufacturer’s protocols. The expression levels of apoptotic proteins were calculated by determining the stained cells and measuring the average in ten microscopic fields (400×).

### 2.3 Statistical analysis

For statistical analysis, the SPSS version 22 (SPSS Incorporated, IL, United States, Chicago) was applied. The *t*-test was used to evaluate the significant differences in the vascular parameters (vessel area and average vessel diameter), MCA and gene expression values. The *p*-value of <0.05 was determined as a significant value.

## 3 Results

### 3.1 *In silico* results

#### 3.1.1 Toxicity of empagliflozin using PASS server

The obtained results from the PASS server were analyzed *via* Pa values (probable to be active). We detected the values of Pa = 0.280 and Pa = 0.199 for EMP, which indicate that the drug is capable of induced vascular and embryo-toxicity.

#### 3.1.2 Structural simulations of apoptotic regulator proteins

The constructed structures of Bax and Bcl-2 proteins were presented in Figures 1C,D. The simulated proteins were analyzed geometrically using Ramachandran plots (Figures 1E,F). Ramachandran results for constructed proteins (Bax and Bcl-2), are presented as follows: 89.4% (Bax) and 90.2% (Bcl-2) of the residues placed in most favored areas, 8.8% (Bax) and 8.4% (Bcl-2) of the residues placed in additional allowed areas and 1.2% (Bax) and 1.4% (Bcl-2) of the residues placed in generously allowed areas. These acquired results reveal that suitable models are simulated.

The molecular features of the simulated Bax consist of; weight = 22,846.60, atoms number = 3,251, amino acids number = 207, number of residues with positive charges (Lys + Arg) = 19, number of residues with negative charges (Glu + Asp) = 20.

The molecular properties of the constructed Bcl-2 including; weight = 21,422.02, atom numbers = 2,954, amino acid numbers of = 194, residues with positive charges (Lys + Arg) = 17, residues with negative charges (Glu + Asp) = 22.

#### 3.1.3 Pocket identification and molecular docking

**FIGURE 2:**
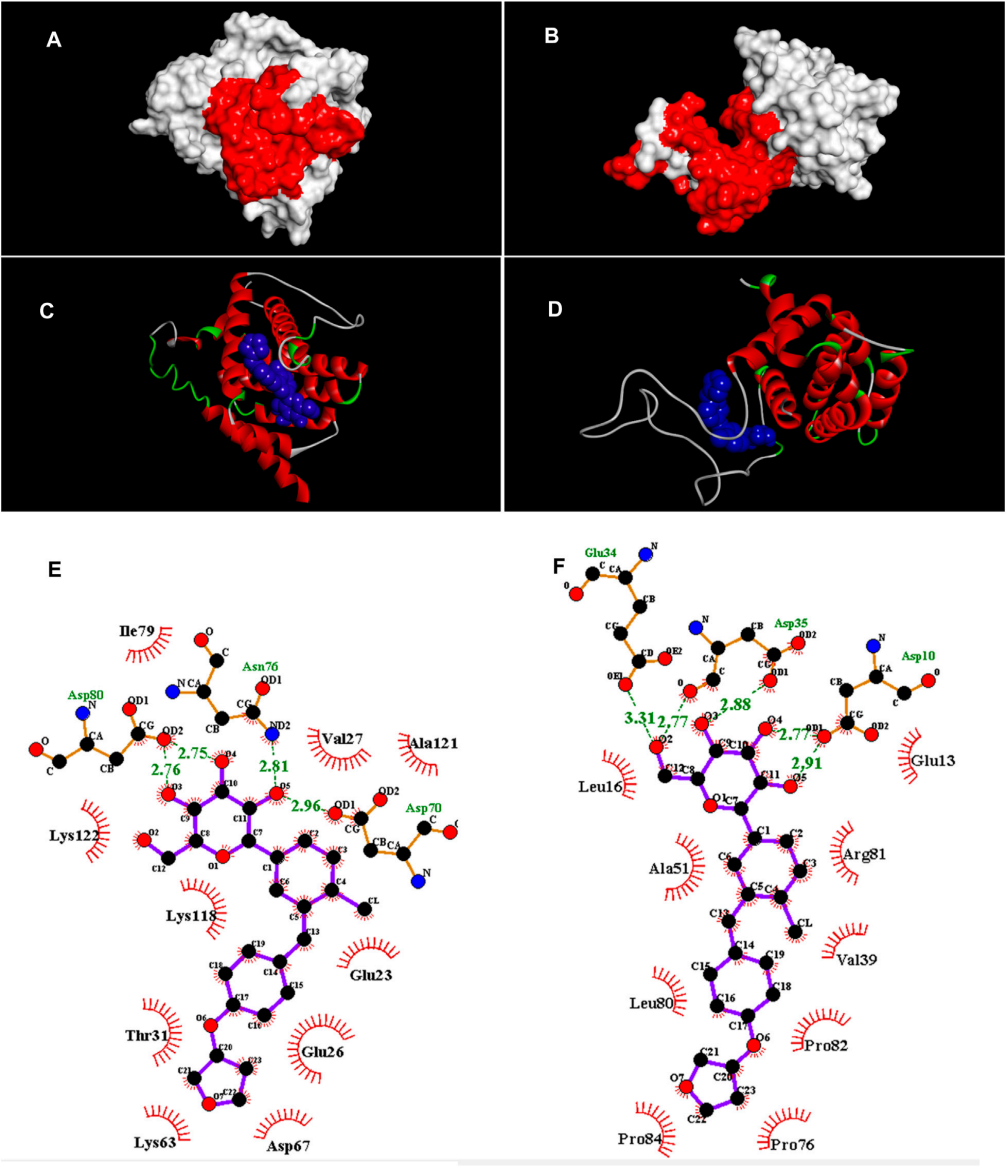
**(A,B)** Pocket sites (red color) in the BAX and Bcl-2, respectively. **(C,D)** Docked structures of EMP with BAX and Bcl-2, respectively. EMP is located in the BAX and Bcl-2 binding sites (blue structure). **(E,F)** Van der Waals and hydrogen interactions (green dots) among EMP, BAX and Bcl-2, respectively. The amino acids that are located in binding sites are illustrated.

The pocket sites on the apoptotic regulator proteins are illustrated in Figures 2A,B. Docking data revealed that EPM was attached to the binding sites of BAX and Bcl-2 (molecular affinity: −7.9 kcal/mol, respectively). Results are presented in Table 1 and Figures 2C,D. As shown in the binding model, EMP was docked to the binding site of BAX with van der Waals interaction and 4 hydrogen bonds between Glu23, Glu26, Val27, Thr31, Lys63, Asp67, Asp70, Asn76, Ile79, Asp80, Lys118, Ala121, and Lys122 (Figure 2E). When investigating for interacted residues among EMP and Bcl-2, we detected that Asp3, Glu6, Leu9, Glu27, Asp28, Val32, Ala44, Pro69, Leu73, Arg74, Pro75, and Pro77 were important for the binding interactions (by van der Waals interaction and 5 hydrogen bonds) (Figure 2F).

**TABLE 1:** Parameters from the interaction between empagliflozin ligand and BAX and Bcl-2 receptors by AutoDock Vina.

Compound	Docking score for empagliflozin (kcal/mol)	Binding interactions	Interacted residues
Bax	-7.9	Van der Waals Hydrogen bonds (4)^a^	Glu23, Glu26, Val27, Thr31, Lys63, Asp67, Asp70, Asn76, Ile79, Asp80,Lys118,Ala12, Lys122
Bcl-2	-7.9	Van der Waals Hydrogen bonds (5)^a^	Asp3, Glu6, Leu9, Glu27, Asp28, Val32, Ala44, Pro69, Leu73, Arg74, Pro75, Pro77

a Number of hydrogen bonds.

#### 3.1.4 Stability of empagliflozin with apoptotic regulator proteins

##### 3.1.4.1 Root-mean-square fluctuation

**FIGURE 3:**
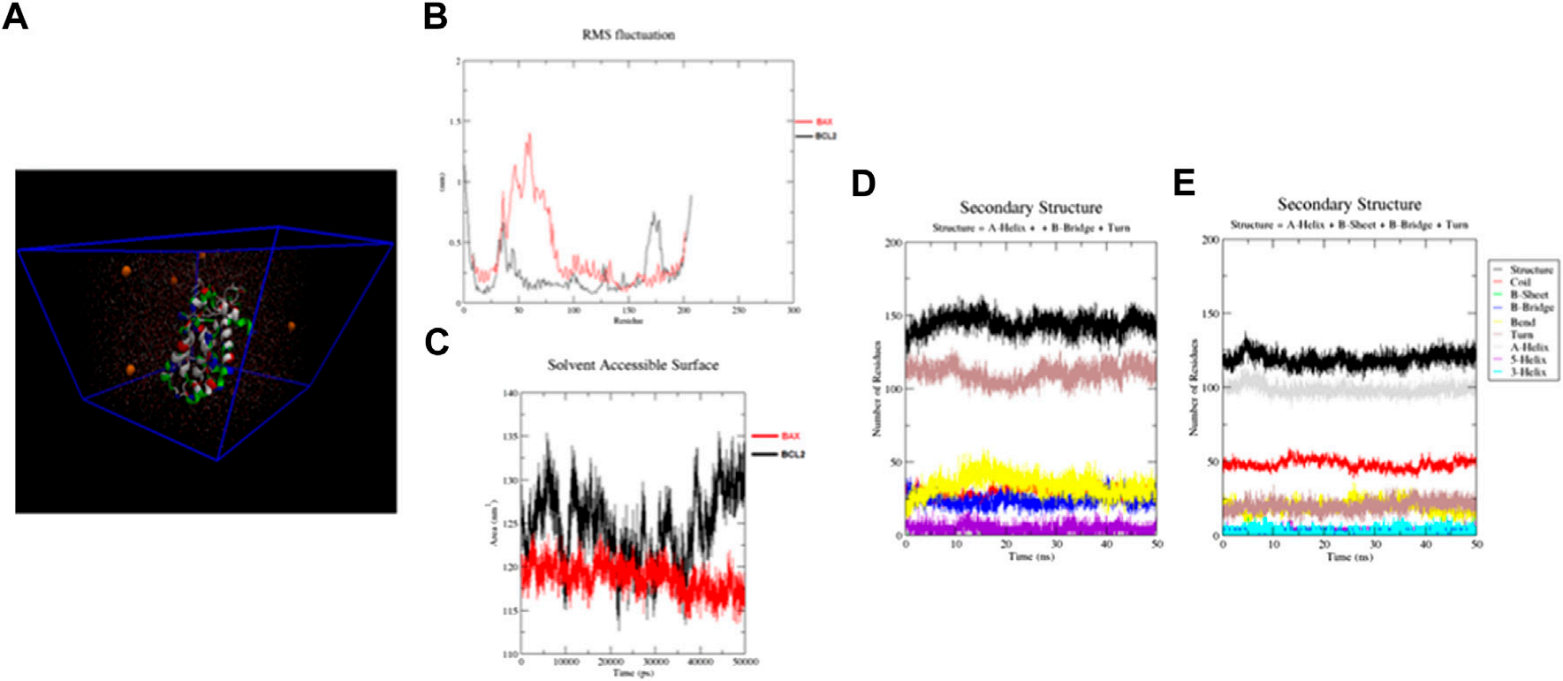
**(A)** Structures solvated in TIP3P water (cyan color) and Na ions (orange color) for molecular dynamics simulations. **(B,C)** RMSF and SASA for constructed structures. By monitoring the RMSF, it is exhibited that the atoms in the EMP/Bax complex exhibit higher fluctuations than the EMP/Bcl-2 complex. The SASA results indicate that the EMP/Bcl-2complex is more stable than the EMP/Bax complex. **(D,E)** The analysis of the secondary structures of EMP interacted with Bax and Bcl-2, respectively.

Detailed analysis of RMSF values is illustrated in Figures 3A,B. The RMSF was applied to monitor each amino acid atom’s mean fluctuation (stability) throughout the MD simulations. The results indicate that the atoms in the EMP/Bax complex exhibit higher fluctuations (0.31 nm) than the EMP/Bcl-2 complex (0.18 nm). This suggests that in the EMP/Bax complex, some amino acid atoms could get away from their normal situations when simulated in the aqueous environment. Therefore, the EMP/Bcl-2 structure is more stable.

##### 3.1.4.2 Solvent accessible surface area

The calculated SASA of the EMP/Bax and EMP/Bcl-2 complexes are presented in Figure 3C. Comparatively, the results indicate that the EMP/Bcl-2complex (124.53 nm^2^) is more stable than the EMP/Bax (118.77 nm^2^) complex in the human body condition. Solvent accessible surface area (SASA) of protein is an important value in structural stability and is considered as hydrophilic, hydrophobic and total values.

##### 3.1.4.3 Secondary structure analysis

The respective secondary structures were depicted, as a function of time, in Figures 3D,E and S5-8**.** Various colors were used to identify the types of secondary structures. Results showed that the overall secondary structure pattern of EMP/Bax and EMP/Bcl-2 were maintained during the MD simulation, though there was a slight deviation in some regions. In EMP/Bax and EMP/Bcl-2 complexes, α-helix, turn and coil were prominent secondary structures observed during simulations.

##### 3.1.4.4 Binding free energy analysis

The energies, which were considered for calculating free energy, are presented in (S9). The estimated binding energies for EMP/Bax and EMP/Bcl-2 complexes were −11.38 and −34.34 kJ/mol, respectively (S9). This shows that the EMP/Bcl-2 fits more comfortably and has a more negative binding free energy compared to EMP/Bax. Both electrostatic and van der Waals interactions were major contributions for binding, while polar salvation was opposing atom binding. However, in the two complexes, it was confirmed that the van der Waals interactions were favor binding energy.

### 3.2 Yolk sac membrane result

#### 3.2.1 Quantification of yolk sac membrane vasculature

**FIGURE 4:**
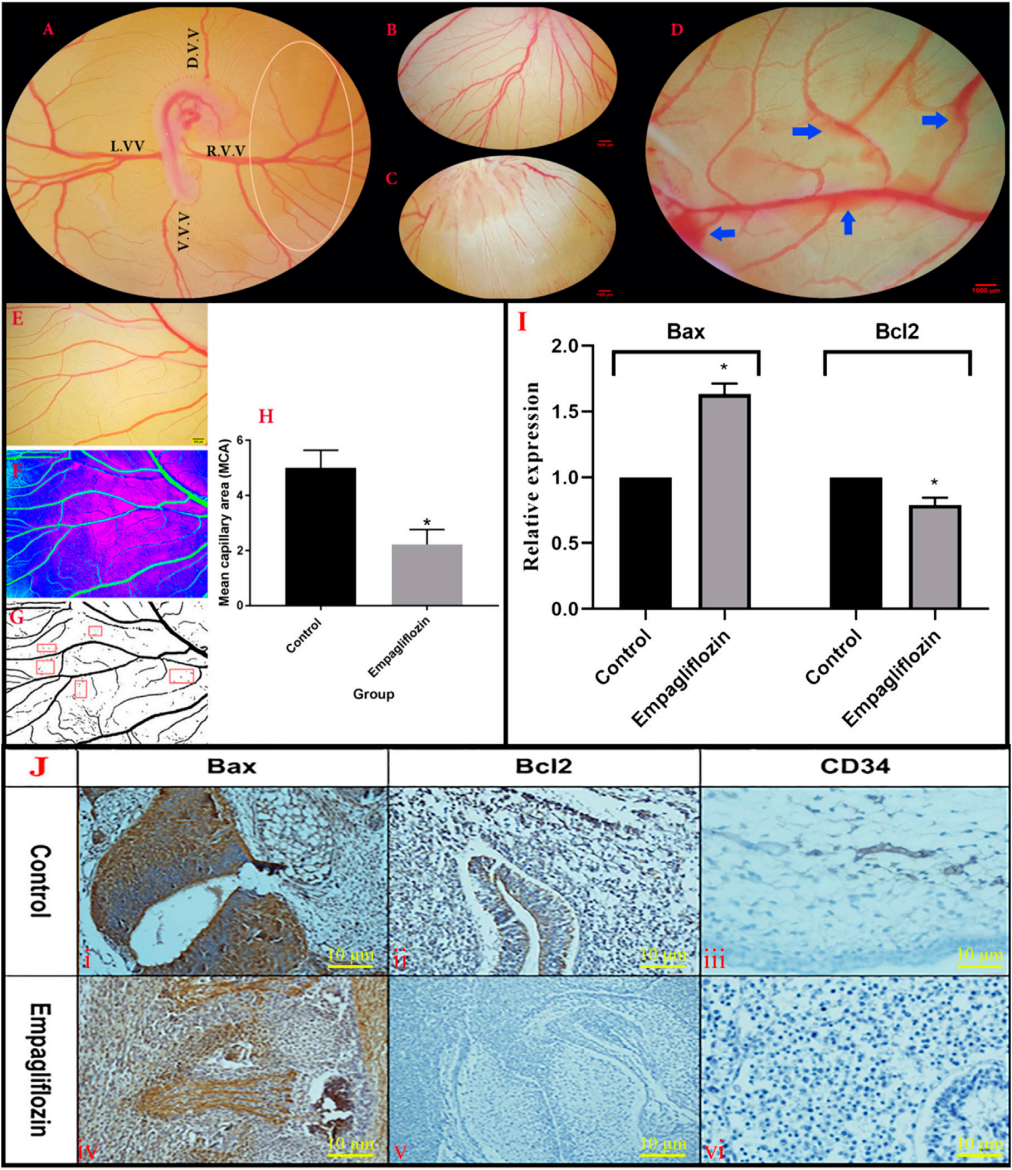
**(A,C)** Morphometric analysis of the YSM vasculature. **(A)** The unique vascular area (Oval) was placed at the right corner of the dorsal and ventral vitelline vein. **(D)**V.V., dorsal vitelline vein; V.V.V., ventral vitelline vein; L.V.V., left vitelline vein; R.V.V., right vitelline vein. **(B)** Control embryo with normal YSM-vasculature. **(C)** Altered pattern of the YSM-vessels in the EMP-treated embryo. **(D)** Vascular bleeding following EMP treatment (blue arrows) **(E–H)** Analysis of mean capillary area (MCA). **(E)** A constant region is chosen from images. **(F)** The threshold of the images was enhanced and converted into the binarized format. **(G)** Five regions (red rectangular) with no-branch vessels were chosen and the percentage of the regions including black pixels (blood) is considered as MCA. **(H)** MCA is reduced in the EMP group compared to the control (error bars display standard error of mean; *p < 0.002, *t*-test). **(I)** Expression level of Bax mRNA is enhanced but the expression level of Bcl-2 is decreased in the EMP-treated group compared to the control. **(J)** The IHC staining. **(JI,JIV)** BAX staining was revealed in the treated group with an increase in their activities of apoptotic changes in the vascular systems and mesenchymal cells. **(JII,JV)** BCL2 staining showed minimal changes of increased activities in the treated group compared with the control group in the vessels and parenchymal cells. **(JIII,JVI)** In the treated group changes in the IHC staining for CD34 of vessels showed a decrease in the intensity of staining along with collapse of them.

Analyzed data from the YSM vessels of the treated embryo are presented in Figure 4A. At the date of quantification, the growth stages of the embryos were 22–24 HH. In the control embryos, the normal patterns of the blood plexuses were around the embryos (Figure 4B). The blood was circulated into the rich vascular plexus and then entered into the sinus terminalis or the vitelline veins. Ultimately, the blood entered into the omphalomesenteric vessel. In the EMP-treated embryos, an altered pattern of the YSM vessels was noticed (Figure 4C). In some cases, vascular bleeding was also noticed on YSM (Figure 4D). The quantification results from the YSM vasculatures showed that the percentages of the vessel area and average vessel diameter were significantly decreased in EMP-treated embryos (%5.39 and 0.055 mm) compared to control (%12.72 and 0.065 mm).

#### 3.2.2 Mean capillary area

The MCA measurement and response to EMP application are illustrated in Figures 4E–H. A significant reduction was noticed in the MCA of the EMP-treated group (control group, 5.01 ± 0.28; treated group, 2.22 ± 0.54; *p* = 0.002).

#### 3.2.3 Gene expression and immunohistochemistry results

The qPCR results revealed enhanced expression of Bax and decreased expression of Bcl-2 genes after EMP treatment (Figure 4I). Furthermore, to verify apoptosis in the YSM vessels, IHC staining was performed for detecting apoptotic cells. As presented in Figure 4J, BAX staining was revealed in the EMP-treated group with an increase in their activities of apoptotic changes in the mesenchymal and vascular systems compared with Bcl-2. In the treated group changes of the IHC staining for CD34 of vessels showed a decrease in the intensity of staining along with collapsed of them.

## 4 Discussion

Various drugs have been applied to control and improve glycemia in patients with type 2 diabetes mellitus. The toxic effect of drugs is of great concern in pregnant women. In this regard, we evaluated some details of the embryo-toxicity of the EMP. Various mechanisms and pathways may be associated with the embryo-toxicity of EMP. In the present investigation, the apoptotic effect of the EMP in embryonic vessels was assessed through *in silico* and *in vivo* studies. In this regard, we discuss various highlights of the findings regarding vascular changes and the interactions of EMP with proteins, which are associated with apoptosis.

In the current investigation, the toxicity of the EMP was assessed *via* the PASS online server. The server was used to predict the toxicity of various chemicals and compounds (Poroikov et al., 2007; Lagunin et al., 2011; Agahi et al., 2020; Salari et al., 2021). Therefore, this server was applied in our study. To confirm our prediction; the toxicity of EMP was also accessed *via in vivo* (YSM) assay. Due to the considerable decreases in vessel area and diameter seen in the YSM vessels, it can be concluded that EMP has a negative effect on the embryonic vasculature.

Determining the toxicity of chemicals requires a preclinical model. The chicken embryo and its extra-embryonic membranes (e.g. YSM and chorioallantoic membrane) are suitable models for this aim due to cost-benefit, reproducibility of results and reduction of legal and ethical aspects. The chick yolk sac consists of the yolk, which provides nutrients, and the YSM, which surrounds the yolk and provides important metabolic functions for the embryo. The YSM is derived from the embryonic midgut and consists of epithelial, mesodermal and ectodermal layers. The YSM is a multifunctional membrane and has vital functions such as erythropoiesis, metabolism, embryonic immunity and nutrient uptake (Wong and Uni, 2021). The YSM and chick’s extra-embryonic membranes models have been used in various researches, and there is a growing repository of data (e.g. anatomical, pharmaceutical, physiological and genetic) that continues to expand the model for toxicological studies (Zhou et al., 2013; Deshpande et al., 2018; Khosravi et al., 2019; Fonseca et al., 2021; Zhang et al., 2022). The chick embryo has also proven to be a manageable and reliable model for evaluating antidiabetic agents (Haselgrübler et al., 2017).

In the current paper, we applied a docking assay to clarify some details about the apoptotic effect of EMP in vessels*.* Currently, docking is considered a useful technique to study the interactions between receptors and ligands, so it is applied in various molecular investigations (Jacob et al., 2017; Kumar et al., 2019; Tavakkoli et al., 2019). It is well known that the Bcl-2 family members are important targets for apoptotic and anti-apoptotic factors (Warren et al., 2019; Trisciuoglio and Del Bufalo, 2021). Here, an important point in our study is the interaction of EMP with Bax and Bcl-2 proteins. Docking results have shown that EMP was bound to the active sites of Bax and Bcl-2 by van der Waals and hydrogen interactions. Our results recommend modulation of Bcl-2 family members through EMP binding. This recommendation should be confirmed by future investigations.

The next highlight of the protein-ligand docking is the affinity between EMP and Bcl-2 family members. Through analyzing the binding data, EMP affinity for Bax and Bcl-2 is predicted to be the same because both have similar scoring energies (−7.9 kcal/mol). Therefore, those proteins can be considered promising targets for the design of anti-apoptotic agents to reduce the deleterious consequences of EMP toxicity in the fetus. Following the docking step, MD simulation was also done to identify the stabilities between EMP, BAX and Bcl-2 proteins inside the human body conditions (aqueous environment, 37.5°C). Herein, dynamic simulations were performed and trajectory data were analyzed *via* Root-mean-square fluctuation (RMSF), solvent accessible surface area (SASA), do_dssp and binding free energy. The results indicated that the EMP/Bcl-2 complex was more stable than EMP/Bax.

In our study, vascular analysis of YSM exhibits that EMP hurts the embryonic vessels. The method used to assess the apoptotic effect of EMP was to calculate the MCA in the obtained images. Until now, this technique has been used in various researches (Seidlitz et al., 2004; Salari et al., 2021).

Another highlight to be explained is the significant change in the expression of Bax and Bcl-2 proteins following EMP treatment. These altered expressions in apoptotic-regulator components can make a link between EMP treatment and vascular defect that was seen in the current study. The pathways or mechanisms by which EMP causes toxic effects on blood vessels is not fully understood but according to our results, it can be suggested that the vascular-toxicity of EMP is associated with the induction of apoptotic-signaling pathways. The IHC results also confirmed the changes in the expressions of Bax and Bcl-2 in the EMP-exposed embryos.

As with any medication, safety considerations limit EMP application during pregnancy. The drug is related to the safety category D in pregnancy. Similarly, there is potential for significant adverse reactions in nursing infants due to the glucose decreasing and volume contraction effects of the EMP (Tuttle et al., 2022). Therefore, it is recommended that the drug should be discontinued while nursing (Halimi and Vergès, 2014; Neeland et al., 2016). The adverse effects of EMP were reported in various research and trials. In this regard, there was an increased incidence of genital mycotic infections in the EMP-treated patients, with mild to moderate severity (Halimi and Vergès, 2014; Neeland et al., 2016). Because EMP causes glucosuria, it is hypothesized that higher levels of glucose in the urine cause fungal overgrowth that leads to infection (Neeland et al., 2016). The natriuretic and diuretic effects of EMP can also exacerbate volume depletion and enhance the risk of hypotension, especially among people with renal failure and the elderly. Recently, there has been increasing concern in the literature about an increased risk of diabetic ketoacidosis, pancreatitis and bone disorders associated with SGLT2 inhibitors (including EMP) (Perkins et al., 2014; Frampton, 2018; Sampani et al., 2020; Dziadkowiec et al., 2021; Bardhi et al., 2022).

In animal investigations, EMP can cross the placenta and result in considerable teratogenic effects (e.g. impaired kidney development and maturation) (Neeland et al., 2016). In experimental studies in dogs and rodents, signs of toxicity were noticed. Most toxicity signs were related to urinary glucose loss and electrolyte imbalances including dehydration, diarrhea, decreased serum glucose, increased protein metabolism, polyuria, glucosuria, and microscopic changes including mineralization in the kidney and some vascular and soft tissues (Bogdanffy et al., 2014; Taub et al., 2015; Knight et al., 2018).

In our research, some other adverse effect of EMP was evaluated, which extend our clinical knowledge of drug toxicity during fetal growth. Herein, we show that the EMP causes vascular defects in embryonic vasculature due to apoptotic activity. This ability can alter the normal growth and development of the fetus; because normal growth of the fetus is associated with normal growth of the embryonic vessels. Currently, there are no controlled human trials of EMP in pregnant women to evaluate its teratogenicity. To our knowledge, there are only rare reports of type 2 diabetic patients who have become pregnant during treatment with EMP (Formoso et al., 2018; Grünert et al., 2022). Those mothers delivered healthy children with no congenital malformations or other injuries. Although our experience is not consistent with the cases reported by Formoso et al. (2018) and Grünert et al. (2022), controlled clinical trials and experiments on larger patient numbers are warranted to verify the safe use of EMP during pregnancy and nursing (Formoso et al., 2018; Grünert et al., 2022).

The EMP that was used in our study was administered at the early stage of the chick embryo development, which is comparable to the first trimester of pregnancy in humans. Therefore, it is suggested that clinicians should limit drug prescription in pregnancy, particularly in the first trimester, since the blood system of the fetus begins to form and expand, and this increases the possibility of vascular disorders. However, it needs further investigations on the human fetus (if ethics committees rule that it is an ethical protocol) to support our results. Ongoing research should also be directed toward identifying which patients are at greatest risk for EMP and toward developing strategies to minimize risk for patients.

## 5 Conclusion

As far as the authors are aware, the current investigation is the first to assess the apoptotic impact of EMP in embryonic vessels by employing the *in silico* and YSM assays. These assays offer promising techniques for investigating the toxic effects of various agents. In the present experiment, we showed that the induction of the apoptotic signal in embryonic vessels is a mechanism, which is related to the embryo-toxicity of EMP. Our acquired data have confirmed that EMP has an adverse impact on the normal expression of apoptotic genes and proteins and has destructive outcomes (e.g. defect of the vascular system). On the other hand, our *in silico* evaluations have also shown that there were significant interactions between EMP, Bax, and Bcl-2. There are various proteins, which are involved in apoptosis. In the current paper, Bax, and Bcl-2 were considered for *in vivo* and *in silico* studies. The EMP may alter various apoptotic proteins and pathways. This provides important recommendation for future research. We believe that the results obtained by our study will improve the clinician’s knowledge about the adverse effects of EMP on the fetus. Taken together, the acquired data permit us to propose that the consumption of EMP must be limited in the pregnancy period or only be applied when its benefits outweigh the risk. Accordingly, the consumption of suitable alternatives must be considered as a goal.

## Data Availability

The datasets presented in this study can be found in online repositories. The names of the repository/repositories and accession number(s) can be found in the article/Supplementary Material.
