# Supplementary material for: Empagliflozin induces apoptotic-signaling pathway in embryonic vasculature: In vivo and in silico approaches via chick’s yolk sac membrane model
Source: Front Pharmacol. 2022 Sep 1;13:970402. doi: 10.3389/fphar.2022.970402 (PMC9474685; doi:10.3389/fphar.2022.970402)
Supplement: Supplementary file 1 [file Table1.DOCX]

**Supplementary**

**S1.** **Input parameter for energy minimization during MD simulation.**

title = Minimization

integrator = steep

emtol = 1000.0

emstep = 0.01

nsteps = 50000

nstlist = 1

cutoff-scheme = Verlet

ns_type = grid

rlist = 1.2

coulombtype = PME

rcoulomb = 1.2

vdwtype = cutoff

vdw-modifier = force-switch

rvdw-switch = 1.0

rvdw = 1.2

pbc = xyz

DispCorr = no

**S2.** **Input parameter for NVT equilibration during MD simulation.**

title = NVT equilibration

define = -DPOSRES

; Run parameters

integrator = md

nsteps = 50000

dt = 0.002

; Output control

nstenergy = 500

nstlog = 500

nstxout-compressed = 500

continuation = no

constraint_algorithm = lincs

constraints = h-bonds

lincs_iter = 1

lincs_order = 4

cutoff-scheme = Verlet

ns_type = grid

nstlist = 20

rlist = 1.2

vdwtype = cutoff

vdw-modifier = force-switch

rvdw-switch = 1.0

rvdw = 1.2

; Electrostatics

coulombtype = PME

rcoulomb = 1.2

pme_order = 4

fourierspacing = 0.16

; Temperature coupling

tcoupl = V-rescale

tc-grps = Protein_JZ4 Water_and_ions

tau_t = 0.1 0.1

ref_t = 300 300

; Pressure coupling

pcoupl = no

; Periodic boundary conditions

pbc = xyz

; Dispersion correction is not used for proteins with the C36 additive FF

DispCorr = no

; Velocity generation

gen_vel = yes

gen_temp = 300

gen_seed = -1

**S3.** **Input parameter for NPT equilibration during MD simulation.**

title = NPT equilibration

define = -DPOSRES

integrator = md

nsteps = 50000

dt = 0.002

nstenergy = 500

nstlog = 500

nstxout-compressed = 500

; Bond parameters

continuation = yes

constraint_algorithm = lincs

constraints = h-bonds

lincs_iter = 1

lincs_order = 4

cutoff-scheme = Verlet

ns_type = grid

nstlist = 20

rlist = 1.2

vdwtype = cutoff

vdw-modifier = force-switch

rvdw-switch = 1.0

rvdw = 1.2

coulombtype = PME

rcoulomb = 1.2

pme_order = 4

fourierspacing = 0.16

; Temperature coupling

tcoupl = V-rescale

tc-grps = Protein_JZ4 Water_and_ions

tau_t = 0.1 0.1

ref_t = 310.65 310.65

pcoupl = Berendsen

pcoupltype = isotropic

tau_p = 2.0

ref_p = 1.0

compressibility = 4.5e-5

refcoord_scaling = com

; Periodic boundary conditions

pbc = xyz ; 3-D PBC

DispCorr = no

gen_vel = no

**S4. The specific primers and reference gene sequences, which applied for qPCR.**

1. Bax (Forward: CCCGAGAGGTCTTTTTCCGAG)

(Reverse: CCAGCCCATGATGGTTCTGAT)

1. Bcl-2 (Forward: AGCGTCAACCGGGAGATGT)

(Reverse: GCATCCCATCCTCCGTTGT)

1. HPRT (Forward: GATGAACAAGGTTACGACCTGGA)

(Reverse: TATAGCCACCCTTGAGTACACAGAG)

1. GAPDH (Forward: CCTCTCTGGCAAAGTCCAAG)

(Reverse: GGTCACGCTGGAAGATA)

**S5.** **The diagram of the secondary structures of EMP with Bax, using DSSP program, during MD simulation.**


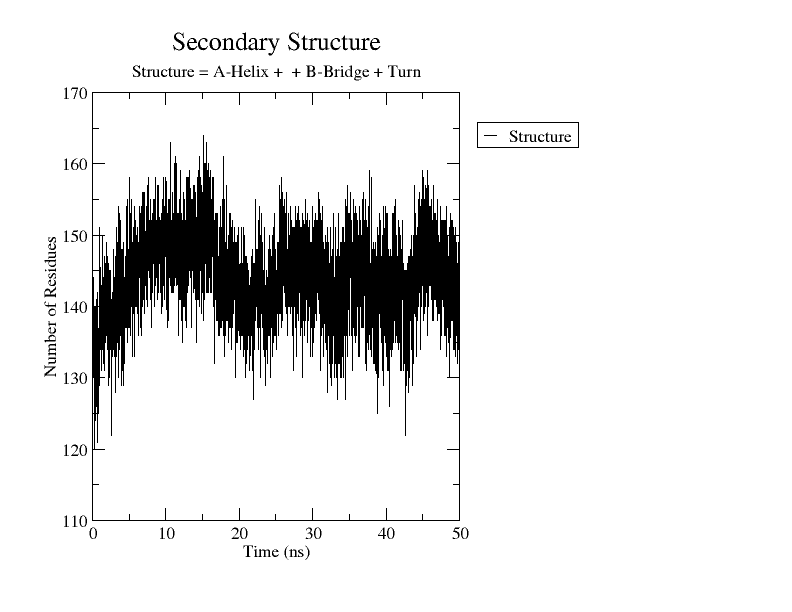


**S6.** **The analysis of the secondary structures of EMP with Bax during MD simulation.**

@ s0 legend "Structure"

@ s1 legend "Coil"

@ s2 legend "B-Bridge"

@ s3 legend "Bend"

@ s4 legend "Turn"

@ s5 legend "A-Helix"

@ s6 legend "5-Helix"

@ s7 legend "3-Helix"

0 149 27 2 25 17 130 5 0

0.01 139 36 2 20 17 120 0 11

0.02 141 33 2 23 17 122 0 9

0.03 141 31 2 24 21 118 0 10

0.04 144 34 2 21 22 120 0 7

0.05 138 36 2 21 20 116 0 11

0.06 135 36 2 19 20 113 5 11

0.07 130 35 2 24 13 115 5 12

0.08 140 31 2 23 19 119 5 7

0.09 144 31 2 24 21 121 0 7

0.1 134 33 2 25 18 114 5 9.

.

.

.

…49.87 145 34 0 24 30 115 0 3

49.88 134 30 0 25 29 105 5 12

49.89 144 27 0 27 30 114 5 3

49.9 148 29 0 21 41 107 5 3

49.91 138 33 0 27 23 115 5 3

49.92 149 28 0 21 36 113 5 3

49.93 143 30 0 22 34 109 5 6

49.94 138 31 0 26 21 117 5 6

49.95 143 34 0 20 25 118 5 4

49.96 138 27 0 27 32 106 5 9

49.97 140 31 0 20 35 105 5 10

49.98 141 30 0 23 28 113 5 7

49.99 144 29 0 25 27 117 5 3

50 143 25 0 30 29 114 5 3

# Totals 720680 147570 152 120959 169816 550712 18883 22114

# SS pr. 0.70 0.14 0.00 0.12 0.16 0.53 0.02 0.02

**S7.** **The diagram of the secondary structures of EMP with Bcl-2, using DSSP program, during MD simulation.**


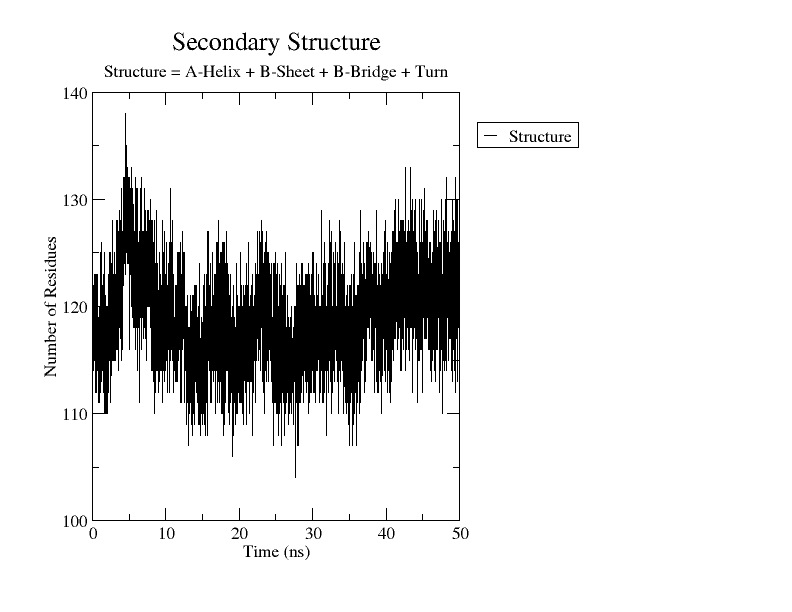


**S8.** **The analysis of the secondary structures of EMP with Bcl-2 during MD simulation.**

@ s0 legend "Structure"

@ s1 legend "Coil"

@ s2 legend "B-Sheet"

@ s3 legend "B-Bridge"

@ s4 legend "Bend"

@ s5 legend "Turn"

@ s6 legend "A-Helix"

@ s7 legend "5-Helix"

@ s8 legend "3-Helix"

0 117 48 0 0 20 16 101 5 3

0.01 123 49 0 0 16 21 102 5 0

0.02 122 44 0 0 24 17 105 0 3

0.03 123 49 0 0 21 21 102 0 0

0.04 119 46 0 0 20 19 100 5 3

0.05 118 48 0 0 19 17 101 5 3

0.06 117 44 0 0 24 18 99 5 3

0.07 119 47 0 0 22 20 99 5 0

0.08 127 43 0 0 20 23 104 0 3

0.09 121 42 0 0 22 20 101 5 3

0.1 115 47 0 0 20 12 103 5 6

0.11 118 44 0 0 23 17 101 5 3

0.12 116 50 0 0 19 16 100 5 3

0.13 115 48 0 0 19 15 100 5 6

0.14 115 51 0 0 19 14 101 5 3

0.15 119 49 0 0 17 20 99 5 3

0.16 117 50 0 0 18 17 100 5 3

.

.

.

49.87 120 51 0 0 14 19 101 5 3

49.88 121 49 0 0 15 21 100 5 3

49.89 123 52 0 0 12 19 104 0 6

49.9 121 52 0 0 14 22 99 0 6

49.91 122 53 0 2 12 23 97 0 6

49.92 126 48 0 0 16 26 100 0 3

49.93 122 49 0 0 19 22 100 0 3

49.94 125 50 0 0 13 27 98 5 0

49.95 130 49 0 2 14 24 104 0 0

49.96 124 51 0 0 18 28 96 0 0

49.97 127 50 0 0 16 25 102 0 0

49.98 126 50 0 0 17 24 102 0 0

49.99 129 50 0 0 14 26 103 0 0

50 126 48 0 2 14 25 99 5 0

# Totals 594702 240151 16 894 97370 100833 492959 20700 12270

# SS pr. 0.62 0.25 0.00 0.00 0.10 0.10 0.51 0.02 0.01

**S9. Binding free energy components for EMP/apoptotic proteins using MM-PBSA method.**

| **Energy components** | **Empagliflozin** | |
| --- | --- | --- |
|  | **Bax** | **Bcl-2** |
| **van der Waals energy (kJ/mol)** | -29.78 | -58.64 |
| **Electrostatic energy (kJ/mol)** | -8.66 | -9.32 |
| **Polar solvation energy (kJ/mol)** | 30.61 | 40.89 |
| **SASA energy (kJ/mol)** | -3.45 | -7.26 |
| **Binding free energy (kJ/mol)** | -11.38 | -34.34 |
|  | | |

**S10. Abbreviations**

| BAX | Bcl2-associated X protein |
| --- | --- |
| Bcl | B-Cell Lymphoma |
| EMP | Empagliflozin |
| H&E | Hematoxylin and eosin |
| IHC | Immunohistochemistry |
| MD | Molecular dynamics |
| YSM | Yolk sac membrane |
