# Supplementary figures and images for: Empagliflozin induces apoptotic-signaling pathway in embryonic vasculature: In vivo and in silico approaches via chick’s yolk sac membrane model
Source: Front Pharmacol. 2022 Sep 1;13:970402. doi: 10.3389/fphar.2022.970402 (PMC9474685; doi:10.3389/fphar.2022.970402)

**Diagrammatic graphic**


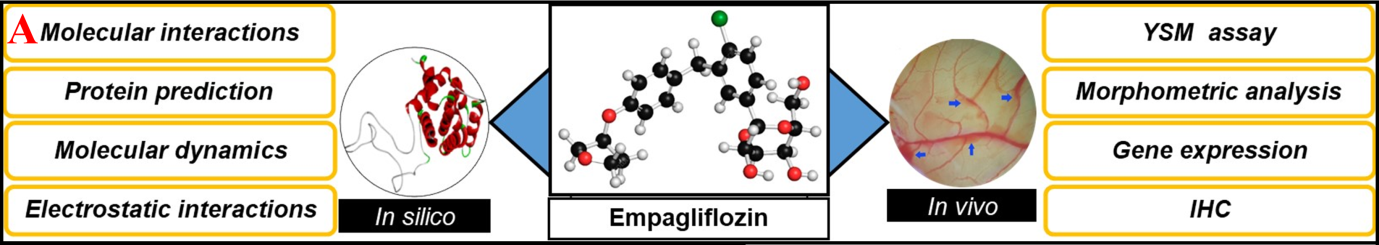

Supplement: Supplementary file 2 [file Table2.DOCX]
